# Herbal medicine formula Huazhuo Tiaozhi granule ameliorates dyslipidaemia via regulating histone lactylation and miR-155-5p biogenesis

**DOI:** 10.1186/s13148-023-01573-y

**Published:** 2023-11-02

**Authors:** Xiangjun Yin, Min Li, Yongzhi Wang, Guifang Zhao, Tao Yang, Yuqing Zhang, Jianbo Guo, Tiantian Meng, Ruolin Du, Honglin Li, Zhe Wang, Jian Zhang, Qingyong He

**Affiliations:** 1 Department of Cardiology, Guang’anmen Hospital, China Academy of Chinese Medical Sciences, Beijing, 100032 China; 2 School of Basic Medical Science, Zhejiang Chinese Medical University, Hangzhou, 310053 China; 3 Shanghai Key Laboratory of New Drug Design, School of Pharmacy, East China University of Science and Technology, Shanghai, 200237 China; 4 LKS Faculty of Medicine, The University of Hong Kong, Pok Fu Lam, Hong Kong China

**Keywords:** Dyslipidaemia, Lactylation, Histone, miR-155-5p, Herbal medicine formula, Huazhuo Tiaozhi granule

## Abstract

**Background:**

Huazhuo Tiaozhi granule (HTG) is a herbal medicine formula widely used in clinical practice for hypolipidaemic effects. However, the molecular mechanisms underlying dyslipidaemia treatment have not been well elucidated.

**Results:**

A significant reduction in the levels of total cholesterol (TC) and low-density lipoprotein cholesterol (LDL-C) was observed in the serum of patients with dyslipidaemia after HTG treatment, without disruption in the levels of aspartate transaminase (AST), alanine transaminase (ALT), urea nitrogen (BUN), and creatinine (Cr). The dyslipidaemia rat model was induced by a high-fat diet and treated with Xuezhikang (0.14 g/kg/d) or HTG (9.33 g crude herb/kg/day) by gavage for 8 weeks. Body weight and liver index were markedly decreased in dyslipidaemic rats after treatment with Xuezhikang or HTG. HTG administration markedly ameliorated hyperlipidaemia by decreasing the levels of TC and LDL-C in serum and hepatic lipid accumulation. In vitro, lipid accumulation in LO2 and HepG2 cells was alleviated by serum treatment with HTG. High lactylation was observed in 198 proteins, including lactylation of histone H2B (K6), H4 (K80). Deep sequencing of microRNAs showed that miR-155-5p was significantly downregulated.

**Conclusions:**

This study demonstrates that HTG is an effective and safe formula for treating dyslipidaemia, which promotes lactylation in hepatocytes, and the retardation of miR-155-5p biogenesis.

**Graphical abstract:**

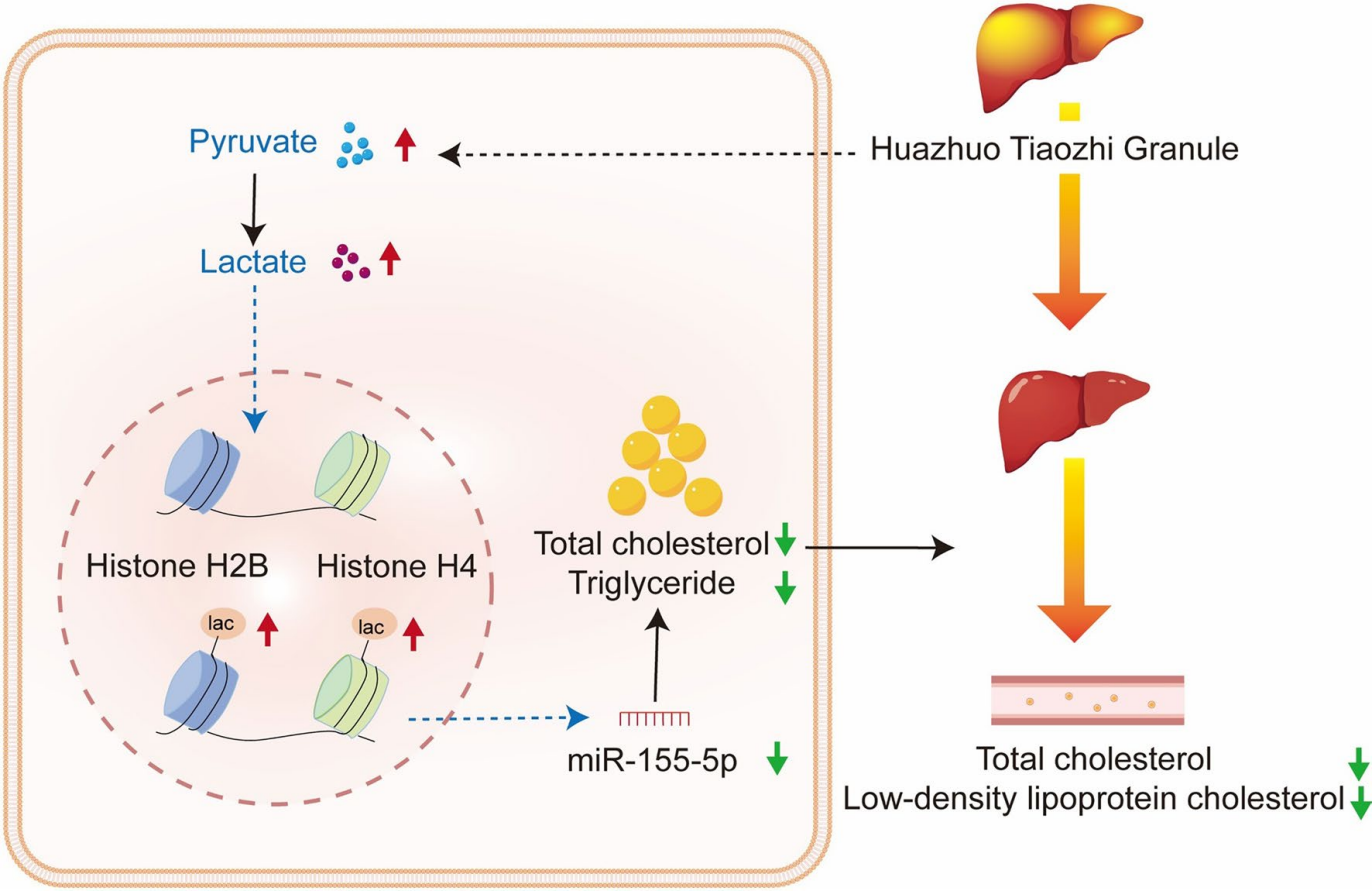

**Supplementary Information:**

The online version contains supplementary material available at 10.1186/s13148-023-01573-y.

## Background

Dyslipidaemia is characterised by increased levels of total cholesterol (TC), triglycerides (TG), and low-density lipoprotein cholesterol (LDL-C), and decreased levels of high-density lipoprotein cholesterol (HDL-C) in the plasma. Hypercholesterolaemia is the most common type of dyslipidaemia, and elevated LDL-C levels are major risk factors for cardiovascular disease, the 8th leading risk factor for mortality in 2019 [1]. Thus, it is important to control plasma lipid concentrations.

Currently, lipid-lowering therapies mainly include statins, ezetimibe, bile acid sequestrants, and pro-protein convertase subtilisin/kexin type 9 inhibitors. Among these drugs, statins are the cornerstone of LDL-C lowering and are widely used in high-income countries which have substantially reduced the number of deaths from cardiovascular disease [2]. However, there are still some problems that cannot be ignored with the use of statins, such as potential harm to the liver and muscle, correlation with the emergence of new-onset diabetes mellitus, cognitive impairment, haemorrhagic stroke, and economic burden [3, 4]. Therefore, there is an urgent need for safe, effective, and affordable lipid-lowering agents.

Traditional Chinese medicine (TCM) has a history of more than 2000 years and plays an important role in the prevention and treatment of various diseases. It has the characteristics of low cost, effectiveness, and fewer side effects, and is widely used in China for treating dyslipidaemia, alone or in combination with western medicine [5–7]. Changes in diet and behaviour are the main causes of dyslipidaemia. From the perspective of the TCM theoretical system, these changes affect the spleen function and promote turbidity. Hence, the method of invigorating the spleen and dissolving turbidity is usually used to treat dyslipidaemia. Huazhuo Tiaozhi granule (HTG) is a representative formula. It was modified from the Zhizhu Pill, which was recorded in the book of *Treaties on the identification of Internal Diseases and External Damages* written by Li Gao from the Jin Dynasty. There are seven Chinese herbs in this prescription: Baizhu, Zhishi, Heye, Shanzha, Danshen, Bixie, and Huzhang. However, the mechanism of action of HTG in treating dyslipidaemia remains unclear.

Lactate is not only an energy source or a by-product of glycolysis, but recent studies have shown that a new post-translational modification, protein lactylation, is derived from lactate [8]. Accumulating evidence indicates that lactate acts as a signalling molecule and mediates gene transcription by lactylation of histones or non-histone proteins. Lactylation plays an important role in lactate metabolism and diseases [9]. It was reported that cellular plasticity is associated with histone lysine lactylation, a key sensor of metabolism [8]. Another study found that lipid accumulation could be improved by regulating fatty acid synthase lactylation [10]. Epigenetic reprogramming-linked lactylation is related to chronic metabolic disease [3, 11].

In this study, we investigated the effect of HTG on dyslipidaemia and explored the potential mechanism by which HTG regulates lipid metabolism in hepatocytes, using mRNA sequencing, microRNA (miRNA) sequencing, and lactylation proteomics. This study provides a novel pharmacological therapy for the treatment of dyslipidaemia and sheds new light on the role of lactylation in regulating miRNA biogenesis.

## Materials and methods

### Preparation of HTG

HTG contains seven Chinese herbs: including *rhizoma atractylodes macrocephalae* Baizhu (12 g), *fructus aurantii immaturus* Zhishi (6 g), *nelumbinis folium* Heye (10 g), *crataegi fructus* Shanzha (12 g)*, salvia miltiorrhiza* Danshen (15 g), *dioscoreae spongiosae rhizoma* Bixie (10 g), and *polygonum cuspidatum* Huzhang (15 g). All herbs were purchased from the Guang’anmen Hospital, China Academy of Chinese Medicine Science. HTG ingredients are mainly obtained via water or ethanol extraction. Briefly, *rhizoma atractylodes macrocephalae, fructus aurantii immaturus, nelumbinis folium, dioscoreae spongiosae rhizoma* were extracted twice in distilled water (1:10, w/v), for 1 h each time; *crataegi fructus* was extracted using a hydro-ethanolic solvent (70/30 ethanol and distilled water, v/v) twice, for 3 h and 2 h, respectively, then it was boiled in distilled water (1:10, w/v) three times, for 3 h, 2 h, and 1 h, respectively; *salvia miltiorrhiza* was extracted using a hydro-ethanolic solvent (80/20 ethanol and distilled water, v/v) twice, for 1.5 h and 1 h, respectively, then it was boiled in distilled water twice (1:10, w/v), for 1.5 h and 1 h, respectively; *polygonum cuspidatum* was boiled in distilled water twice, for 1 h each time, then hydro-ethanolic solvent (95/5 ethanol and distilled water, v/v) was added and allowed to stand for 12 h, the supernatant was collected. The filtered solutions were concentrated with a rotary evaporator at 60 °C, then the concentrates were evaporated to dryness. Finally, the extracts were stored at 4 °C for further experiments.

### Clinical participants and data collection

A total of 12 participants diagnosed with dyslipidaemia were enrolled in accordance with the Guidelines for the Prevention and Treatment of Dyslipidemia in Chinese Adults (2016, revised edition). Meanwhile, they met the TCM diagnosis of spleen deficiency and phlegm-blood stasis syndrome, according to the Guidance Principle of Clinical Study on New Drugs of Traditional Chinese Medicine (2002, revision edition). Patients were orally administrated with HTG (equivalent to 80 g of crude drug per day) provided by Jiangyin Tianjiang Pharmaceutical Co. Ltd. (Jiangyin, Jiangsu Province, China) for 8 weeks. During the treatment period, they were advised to follow a balanced and nutritious diet and exercise. Before and after treatment, blood was collected and lipid levels (TC, TG, LDL-C, and HDL-C) were detected. The safety of HTG was evaluated using aspartate transaminase (AST), alanine transaminase (ALT), urea nitrogen (BUN), and creatinine (Cr). This study was conducted in accordance with the principles of the Declaration of Helsinki and was approved by the Ethics Committee of Guang’anmen Hospital of the China Academy of Chinese Medicine Science.

### Animals and dyslipidaemia model establishment

130–150 g male Wistar rats were purchased from Beijing Vital River Laboratory Animal Technology Co., Ltd. All animals were housed in the animal room of Guang’anmen Hospital, Chinese Academy of Traditional Chinese Medicine, under specific pathogen free (SPF) conditions, a 12 h light/dark cycle, and had access to food and water ad libitum. All procedures were performed in accordance with the guidelines and regulations of Guang’anmen Hospital.

The dyslipidaemia model was established prior to drug intervention. Wistar rats were fed a high-fat diet (78.8% basic diet, 1% cholesterol, 10% egg yolk powder, 10% pig fat, 0.2% sodium cholate) which was provided by Beijing HFK Bioscience Co., Ltd. (Beijing, China) for 4 weeks. Blood samples were collected from all rats via the retro-orbital venous plexus, and serum TC, TG, HDL-C, and LDL-C levels were measured using an automatic biochemical analyser (Beckman Coulter, Inc., Brea, USA).

### Experimental design

After 1 week of adaptive feeding, 24 rats were weighed and randomly divided into four groups (*n* = 6 per group): normal-fat diet, high-fat diet, xuezhikang, and HTG. Rats in the normal-fat diet group were fed a basic diet (Beijing HFK Bioscience Co., Ltd., Beijing, China), whereas the other rats were fed a high-fat diet for 12 weeks. Fifth week onwards, the animals were gavaged with different drugs suspended or dissolved in 0.5% carboxymethyl cellulose-Na (CMC-Na) twice daily for 8 weeks. The details were as follows: (a) Normal-fat diet group (NFD): received 0.5% CMC-Na 20 mL/kg/d; (b) High-fat diet group (HFD): received 0.5% CMC-Na 20 mL/kg/d; (c) Xuezhikang group (XZK): received Xuezhikang capsule (Beijing WBL Peking University Biotech Co., Ltd., Beijing, China) 0.14 g/kg/d; (d) HTG group (HTG): received Huazhuo Tiaozhi granule 9.33 g crude herb/kg/d.

At the end of the experiment, all the rats were fasted for 12 h and anaesthetised with sodium pentobarbital (45 mg/kg, intraperitoneal injection). Blood samples were collected from abdominal aorta and kept at room temperature for 2 h, then centrifuged at 3000 rpm for 15 min at 4 °C to obtain the serum. The liver tissues were also harvested, they were washed with 0.9% saline solution and weighed, cut into four pieces, two pieces were quickly frozen in liquid nitrogen and then stored at − 80 °C, two pieces were fixed in 10% neutral-buffered formalin.

### Determination of body weight and liver index

The body weights (g) of the rats in all groups were measured weekly. At the end of the experiment, the rats were anaesthetised, abdominal aortic blood was sampled, the liver was quickly removed and weighed, and the liver index was calculated as follows: liver index = liver weight/body weight × 100.

### Biochemical detection

After 8 weeks of pharmacological intervention, the concentrations of serum lipids (TC, TG, HDL-C, and LDL-C) and liver function (AST and ALT) were measured using an automatic biochemical analyser (Beckman Coulter, Inc., Brea, USA).

### Liver histological analysis

The liver tissues were fixed in 10% neutral-buffered formalin for 48 h, dehydrated in ascending ethanol grades, and embedded in paraffin. The samples were sliced into 5 μm and stained with haematoxylin and eosin (H&E). To observe the accumulation of lipid droplets, the fixed tissues were dehydrated with 30% sucrose for 24 h and then frozen and sliced into 10 μm slices. Frozen sections were stained with Oil Red O (Solarbio Science & Technology Co., Ltd., Beijing, China). Finally, histological changes in the liver tissues were observed under a light microscope (Olympus, Tokyo, Japan).

### Preparation of medicated serum

Twenty Sprague–Dawley rats (200–220 g) were randomly divided into two groups (10 rats per group): the control group (with the same volume of 0.5% CMC-Na) and the other group was administered HTG (9.33 g crude herb/kg/d) via gastric perfusion once a day for 7 d. Then blood was taken from abdominal aortic 1 h after the final administration, kept at room temperature for 2 h, centrifuged at 3000 rpm for 15 min at 4 °C to obtain the medicated serum. The serum was inactivated in water at 56 °C for 30 min, then stored at − 80 °C. It was filtrated with a 0.22 μm cellulose acetate membrane before use.

### Effects of HTG on lipid levels in LO2 and HepG2 cells

The human liver cell lines LO2, HepG2 were purchased from iCell Bioscience Inc (Shanghai, China), and cultured in Dulbecco’s modified Eagle’s medium (DMEM, gibco, USA) supplemented with 20% foetal bovine serum (FBS, Biological Industries, Israel), 10% FBS, respectively, 100 U/ml of penicillin, and 100 µg/ml of streptomycin (gibco, USA) at 37 °C and 5% CO_2_. A hyperlipidaemia model of LO2 or HepG2 cells was established using free fatty acids (FFA, oleate and palmitate, 2:1; Sigma-Aldrich, USA) at a final concentration of 1 mM for 24 h, while the control group was incubated in normal medium. Cells were seeded at a density of 250,000 cells/well in 6-well plates. They were allowed to attach overnight prior to HTG-medicated serum for 24 h and then exposed to FFA during drug treatment. After treatment, the concentrations of TC, TG, lactate, and pyruvate in the cells were determined using commercial kits (Jiancheng Bioengineering Institute, Nanjing, China). The cells were fixed in 10% neutral-buffered formalin for 15 min and stained with Oil Red O.

### Analysis of lactylation proteomics

Lactylation proteomics was performed by Jingjie PTM BioLabs (Hangzhou, China). Briefly, the Proteins were extracted from cell samples using a high-intensity ultrasonic processor (Scientz, Ningbo, China) in lysis buffer (8 M urea, 1% protease inhibitor cocktail), digested with trypsin, and the tryptic peptides were labelled with their respective TMT reagents (Thermo Fisher Scientific, MA, USA). To enrich modified peptides, tryptic peptides were dissolved in NETN buffer (100 mM NaCl, 1 mM EDTA, 50 mM Tris–HCl, 0.5% NP-40, pH 8.0) and incubated with prewashed antibody beads (Jingjie PTM BioLabs, Hangzhou, China) at 4 °C overnight with gentle shaking. The resulting peptides were desalted using C18 ZipTips (Millipore, MA, USA) for liquid chromatography coupled with tandem mass spectrometry (LC–MS/MS) analysis. The tryptic peptides were loaded onto a home-made reversed-phase analytical column (25-cm length, 75 μm i.d.) and separated on an EASY-nLC 1200 UPLC system (Thermo Fisher Scientific, MA, USA). The separated peptides were analysed using Q ExactiveTM HF-X (Thermo Fisher Scientific, MA, USA) with a nano-electrospray ion source. The resulting MS/MS data were processed using the MaxQuant search engine (v.1.6.15.0). Gene Ontology (GO) annotation, domain annotation, subcellular localisation, Kyoto Encyclopedia of Genes and Genomes (KEGG) pathway enrichment, and COG/KOG functional classification were performed.

### Seahorse XF glycolytic rate assay

The experiment was performed according to the instructions of the XF Glycolytic Rate Assay Kit instructions (Agilent, Santa Clara, CA, USA). Briefly, LO2 cells were plated at 15,000 cells/well in 96-well Seahorse XF 96 assay plates overnight before exposure to FFA (1 mM) and drug treatment for 24 h. The extracellular acidification rate (ECAR) was measured using an XF 96 flux analyser (Agilent, Santa Clara, CA, USA). On the day of this assay, the culture medium was changed to unbuffered DMEM (DMEM supplemented with 25 mM glucose and 10 mM sodium pyruvate; pH 7.4) and the cells were incubated at 37 °C in a non-CO_2_ incubator for 1 h. LO2 cells were stimulated with rotenone, antimycin A (0.5 μM) and 2-DG (50 mM). Eight independent experiments were performed. ECAR was automatically calculated and recorded using Seahorse XFe-96 software.

### miRNA-seq and data analysis

Total RNA was isolated using the TRIzol reagent (Invitrogen, Carlsbad, CA, USA) and purified using the RNeasy Mini Kit (Qiagen, Carlsbad, USA) according to the manufacturer’s instructions. RNA quality and quantity were measured using Nanodrop ND-1000, and gel electrophoresis was performed to determine RNA integrity. After quality control, the miRCURY™ Hy3™/Hy5™ Power labelling kit (Exiqon, Vedbaek, Denmark) was used according to the manufacturer’s guideline for miRNA labelling. Then the Hy3™-labelled samples were hybridised on the miRCURYTMLNA Array (v.18.0) (Exiqon, Vedbaek, Denmark) according to the array manual. The slides were scanned using an Axon GenePix 4000 B microarray scanner (Axon Instruments, Foster City, CA, USA). The scanned images were imported into GenePix Pro 6.0 software for grid alignment and data extraction. Replicated miRNAs were averaged and miRNAs intensities ≥ 30 in all samples were chosen for calculating the normalisation factor by median normalisation. Differentially expressed miRNAs were identified through fold-change (> 2 or < 0.5) and *p* values (< 0.05).

### RNA-seq

Total RNA was extracted from the liver tissue of dyslipidaemic C57BL/6 J mice using TRIzol reagent (Invitrogen, Carlsbad, CA, USA) and purified using the RNeasy mini kit (Qiagen, Carlsbad, USA). RNA quality and quantity were assessed using a Bioanalyzer 2100 and RNA 6000 Nano LabChip Kit (Agilent, Santa Clara, USA). For library construction, we selected high-quality RNA samples with an RIN > 7.0. The mRNA was purified using Dynabeads Oligo (dT) (Thermo Fisher Scientific, MA, USA). The fragmented RNA was reverse transcribed into cDNA using SuperScript II Reverse Transcriptase (Invitrogen, Carlsbad, CA, USA). To generate U-labelled second-stranded DNAs, Escherichia coli DNA polymerase I, RNase H, and a dUTP solution were used. Adapters with T-base overhangs were ligated to the A-tailed fragmented DNA, and size selection was performed using AMPureXP beads. After treatment with the heat labile UDG enzyme, PCR amplification of the ligated products was performed. The final cDNA library had an average insert size of 300–50 base pairs (bp). Paired-end sequencing (PE150) was conducted on an Illumina NovaSeq 6000 platform following the recommended protocol provided by the vendor.

### Quantitative real-time polymerase chain reaction (qRT-PCR)

The extracted RNA was reverse transcribed to cDNA using the miRNA First-Strand cDNA Synthesis Kit (ComWin Biotech Co., Ltd., Beijing, China) or the riboSCRIPT Reverse Transcription Kit (RiboBio, Guangzhou, China) according to the manufacturer’s instructions. qRT-PCR was performed using a miRNA Real-time PCR Assay Kit (ComWin Biotech Co., Ltd., Beijing, China) or a Bulge-Loop^TM^ miRNA qRT-PCR Kit (RiboBio, Guangzhou, China) according to the manufacturer’s instructions. The PCR procedure was as follows: 95 °C for 10 min, 95 °C for 15 s, and 60 ℃ for 1 min × 40 cycles. The miRNA levels were calculated with the 2^−△△Ct^ method and normalised to U6. The primers were shown in Additional file 1: Table S1.

### Cell transfection for miR-155-5p mimics

Chemical mimics of miR-155 (RiboBio, Guangzhou, China) were used to upregulate the expression level of miR-155. Mimics were transfected into LO2 cells using Lipo8000 Reagent (Beyotime, Shanghai, China) for 48 h. After qRT-PCR analysis of the transfection efficiency, LO2 cells were exposed to FFA- or HTG-medicated serum for subsequent experiments.

### Pull down

The cells were lysed after HTG treatment using lysis buffer (Thermo Fisher Scientific, MA, USA). Then the cell lysates were pulled down by protein A/G agarose (Beyotime, Shanghai, China) and incubated with the primary antibody against L-Lactyl Lysine Rabbit pAb (Jingjie PTM BioLabs, Hangzhou, China) overnight at 4 °C. After elution from beads, the protein was added with sodium dodecyl sulfate (SDS) buffer at 100 °C for 5 min.

### Western blot analysis

Following the quantitative concentration of total protein extracted from the liver tissue or cells, a loading buffer was added for high-temperature denaturation. Subsequently, the chilled protein samples were stored in different containers at –20 °C to prevent repeated freeze–thaw cycles. The protein samples were subjected to electrophoresis on SDS gels and transferred to appropriately sized nitrocellulose membranes soaked in a transfer buffer. To block nonspecific binding, the blots were incubated with 5% skim milk. Primary antibodies against histone H2B (Proteintech, Wuhan, China), histone H4 (Jingjie PTM BioLabs, Hangzhou, China), and α-tubulin (Proteintech, Wuhan, China) were applied to the blots overnight. After washing with tris-buffered saline containing 0.05% Tween-20 (TBST), the protein bands were incubated with a secondary antibody (Cell Signalling Technology, Boston, MA, USA) at room temperature for 1 h. The enhanced ECL immunoblotting system (Tanon, Shanghai, China) was utilised to detect the specific antibody fluorescence density of the protein bands, and ImageJ (NIH, Bethesda, MD, USA) was employed for analysis and statistical evaluation.

### LC–MS/MS analysis of HTG

The LC–MS/MS analysis was performed using a U3000 UHPLC system (Thermo Scientific, MA, USA) coupled with a TripleTOF5600 + mass spectrometer (AB SCIEX™). Components of HTG were separated on a 150 × 2.1 mm BEH C18 column with 1.7 μm particle size (Waters, MA, USA). The column temperature was 35 °C. The mobile phase was a mixture of 0.1% formic acid (A) and acetonitrile (B) delivered at a speed of 0.3 mL/min. The gradient elution was performed as follows: 0–8 min (A: 95%, B: 5%), 8–10 min (A: 50%, B: 50%), 10–12 min (A: 5%, B: 95%), 12–15 min (A: 95%, B: 5%). The mass spectrometer was equipped with an electrospray ionisation (ESI) source and operated in the positive and negative modes. The ESI conditions were performed as follows: ion source gas 1: 50 psi, ion source gas 2: 50 psi, curtain gas: 25 psi, source temperature: 500 °C (positive mode) and 450 °C (negative mode), ion sapary voltage floating (ISVF): 5500 V (positive mode) and 4400 V (negative mode), TOF MS scan range: 100–1200 Da. Secondary mass spectrometry was acquired using information dependent acquisition (IDA) with high sensitivity mode, declustering potential: ± 60 V, collision energy:35 ± 15 eV.

### Statistical analysis

All data are shown as mean ± standard deviation and were analysed using SPSS software (version 20.0; SPSS Inc., Chicago, USA). Comparisons between the two groups were performed using Student’s *t*-test or nonparametric rank sum tests. Multiple comparisons were performed using one way analysis of variance (ANOVA) or nonparametric rank sum tests. *p* values < 0.05 were considered statistically significant.

## Results

### HTG protected against dyslipidaemia

The chemical compounds in HTG were detected using LC–MS/MS and aligned to databases, including MassBank, RIKEN MSn spectral database for phytochemicals (ReSpect), and Global Natural Products Social Molecular Networking (GNPS). The parameters for database matching included peak detection, alignment, and identification settings. A total of 120 chemical compounds (total score ≥ 80) were detected and 10 compounds (total score ≥ 91.5) were listed in Table 1.

**Table 1:** Identification of chemical compounds in HTG

No	TR/min	Adduct	Total score	Formula	Compound
1	5.66	[M−H]−	96.9	C_21_H_20_O_12_	Hyperoside
2	5.18	[M−H]−	95.2	C_26_H_28_O_16_	Peltatoside
3	6.53	[M−H]−	94.6	C_14_H_12_O_3_	Trans-resveratrol
4	5.48	[M+H]+	94.6	C_15_H_10_O_7_	Quercetin
5	9.82	[M−H]−	93.9	C_15_H_10_O_5_	Emodin
6	5.88	[M+H]+	93.5	C_15_H_10_O_6_	Kaempferol
7	6.25	[M−H]−	93.4	C_18_H_16_O_8_	Rosmarinic acid
8	6.13	[M−H]−	92.4	C_25_H_24_O_12_	3,4-di-O-caffeoylquinic acid
9	2.58	[M−H]−	92.1	C_7_H_6_O_3_	Protocatechuic aldehyde
10	4.20	[M−H]−	91.9	C_15_H_14_O_6_	Catechin

**Fig. 1:**
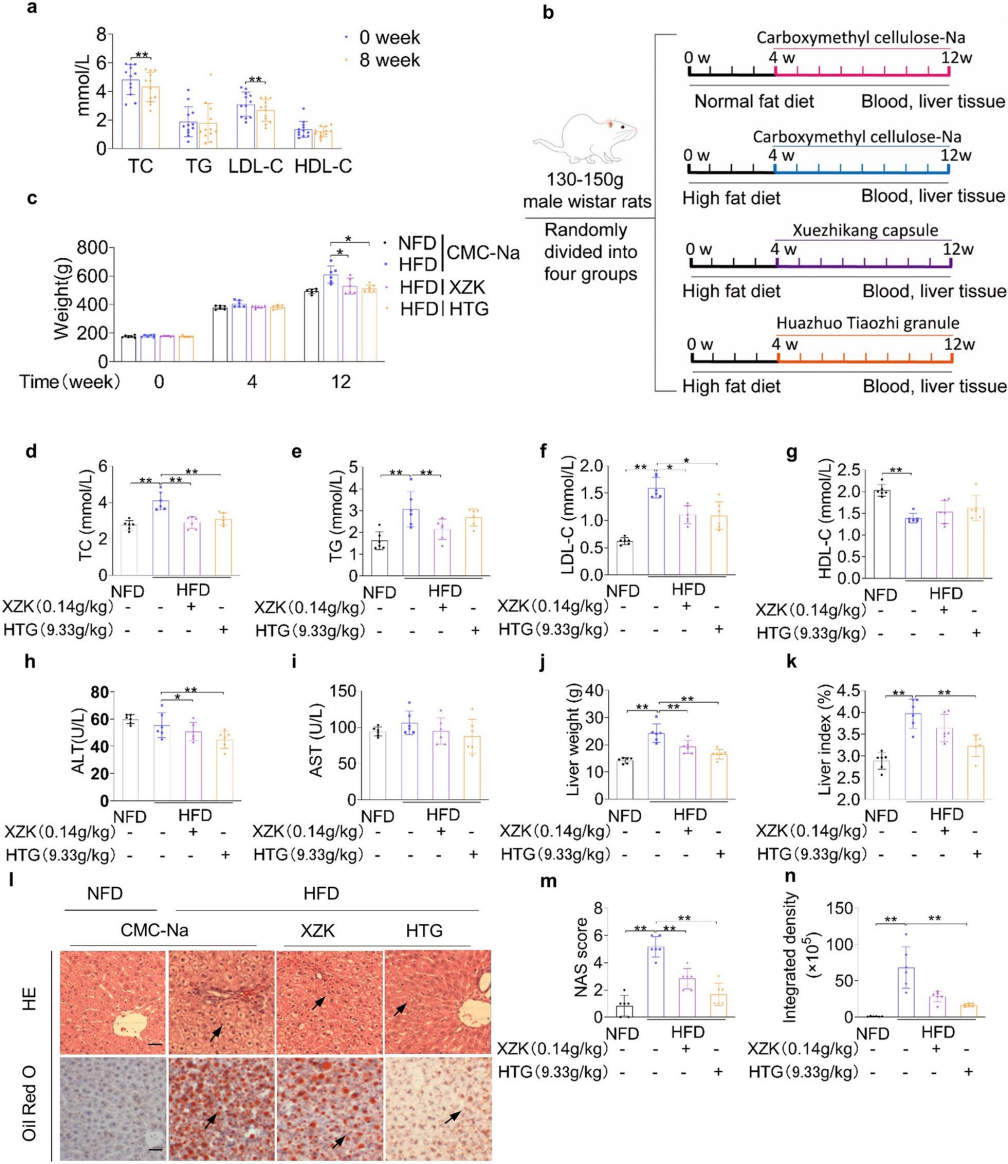
Effects of HTG on lipid profiles. **a** TC, TG, LDL-C, HDL-C levels of dyslipidaemia patients after 8 weeks HTG treatment. **b** Overview of the experimental design in Wistar rats. **c** Body weight of rats at different time points (0, 4, 12 weeks). **d** TC level in serum after HTG treatment for 8 weeks. **e** TG level in serum after HTG treatment for 8 weeks. **f** LDL-C level in serum after HTG treatment for 8 weeks. **g** HDL-C level in serum after HTG treatment for 8 weeks. **h** AST levels in serum. **i** ALT levels in serum. **j** Liver weight of rats after HTG treatment for 8 weeks. **k** Liver index of rats after HTG treatment for 8 weeks. **l, m, n** Histological changes in rats after HTG treatment for 8 weeks. Scale bars, 100 μm. *p < 0.05, **p < 0.01, versus HFD group

We evaluated the efficacy and safety of HTG in treating dyslipidaemia with a treatment period of 8 weeks and a total of 12 participants. The baseline characteristics of the patients were shown in Additional file 2: Table S2. HTG significantly reduced the levels of TC and LDL-C without disrupting serum TG and HDL-C levels (Fig. 1a). The levels of ALT, AST, BUN and Cr remained within normal ranges after treatment (Additional file 3: Table S3).

Rats were fed a high-fat diet to establish a dyslipidaemia model and the effects of HTG and the positive control, XZK were evaluated. The body weight of rats in the HTG group was significantly lower than that of rats in the model group fed a high-fat diet for 12 weeks (Fig. 1b-c). After treatment for 8 weeks, HTG administration significantly reduced the levels of TC and LDL-C in the serum, which was consistent with the results in the human samples (Fig. 1d–g). HTG treatment significantly decreased ALT levels, whereas drug intervention had no significant effect on AST levels (Fig. 1h–i). The liver weight and index of the rats in the XZK and HTG groups were lower than those in the HFD group (Fig. 1j–k). H&E staining showed that HTG treatment protected against the disordered arrangement of hepatocytes, infiltration of inflammatory cells, and hepatic steatosis. HTG administration markedly reduced the accumulation of lipid droplets in the livers of rats fed a high-fat diet, as detected by Oil Red O staining (Fig. 1l–n).

### The treatment of HTG enhanced lipid metabolism in fatty liver in mice

**Fig. 2:**
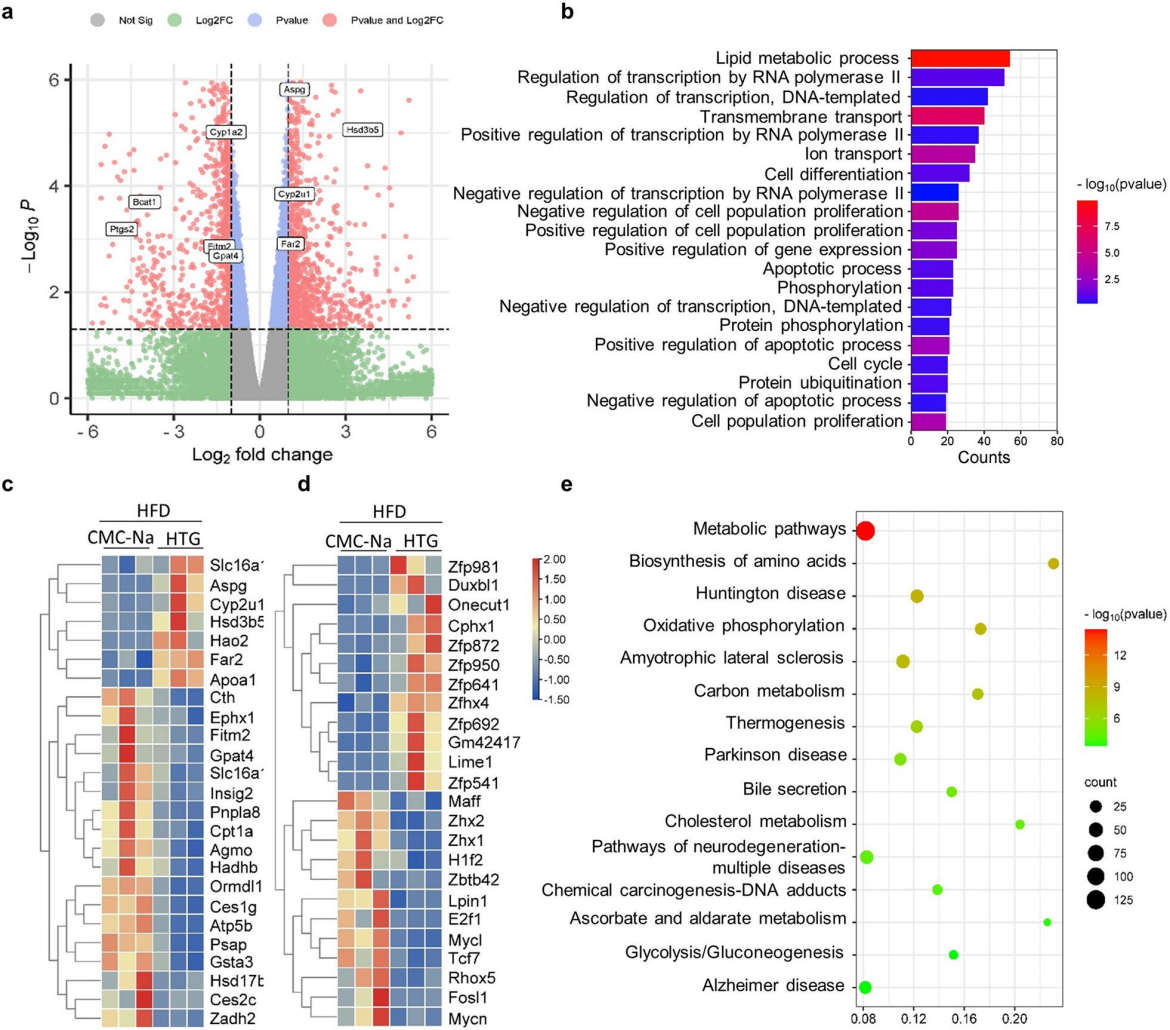
Screening of different expressed genes (DEGs) in liver tissues of dyslipidaemia mouse with HTG treatment or not. **a** Volcano plots. **b** GO analysis shows the biological process of DEGs **c** The heatmap plot of DEGs (top 26) enriched in lipid metabolic process. **d** Heatmap plot of DEGs (top 25) enriched in regulation of transcription by RNA polymerase II. **e** KEGG pathway

Volcano plots of the RNA deep sequencing showed that the RNA expression of 462 genes were increased and that of 580 genes were decreased, respectively (Fig. 2a). GO analysis showed that the biological processes (BP) of lipid metabolism in the fatty liver were upregulated in HTG-treated mice (Fig. 2b–d). This metabolic pathway was enhanced in the HTG group, as confirmed by KEGG analysis (Fig. 2e).

### The treatment of HTG attenuated lipid accumulation and enhanced compensatory glycolysis in vitro

**Fig. 3:**
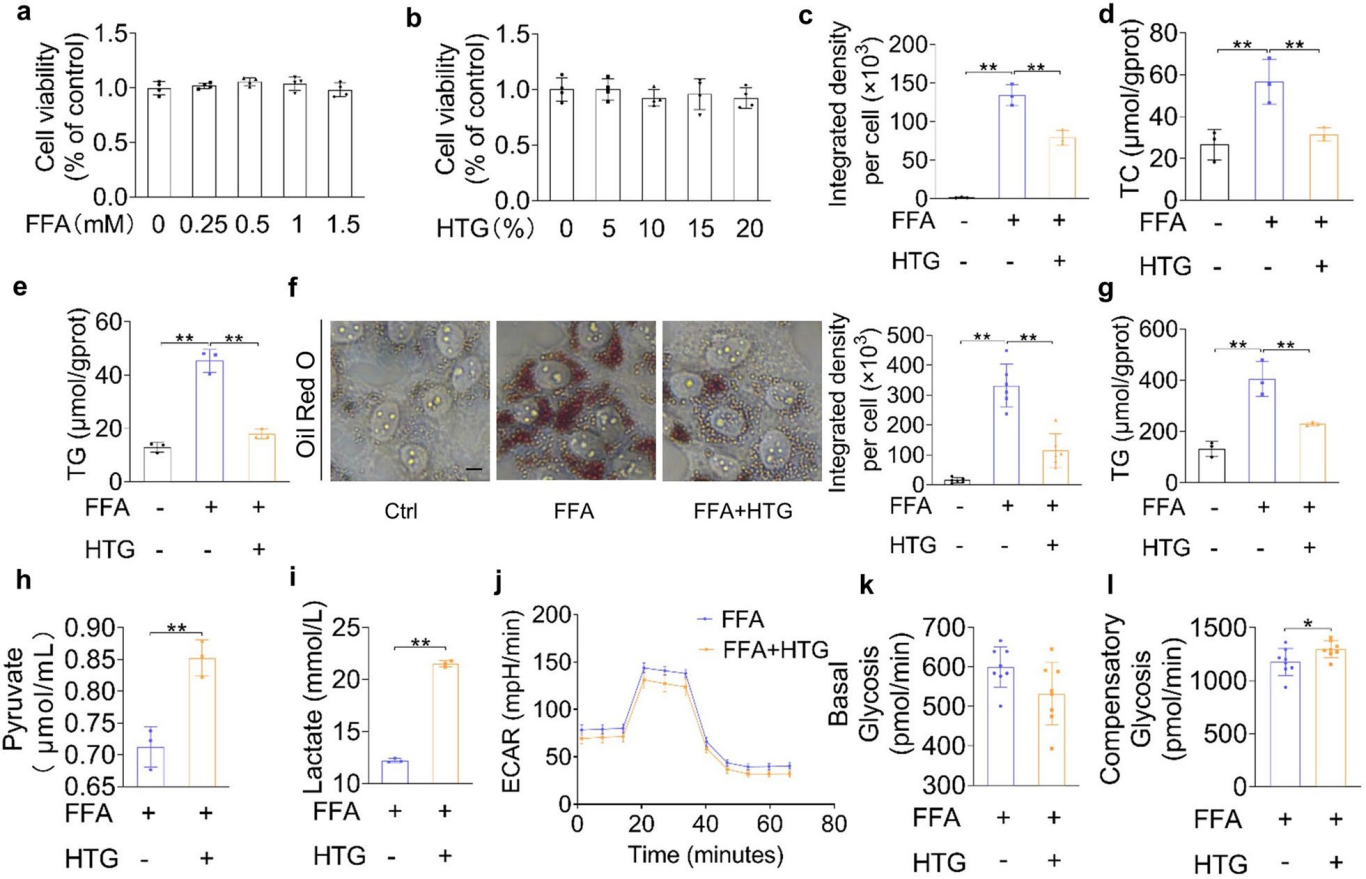
Effects of HTG on liver cells stimulated with FFA (free fatty acids). **a** Cell viability of LO2 cells stimulated with different concentrations of FFA (0, 0.25, 0.5, 1, 1.5 mM) for 24 h. **b** Cell viability of LO2 cells after HTG treatment (0%, 5%, 10%, 15%, 20%) for 24 h. **c** Integrated density per LO2 cell after red oil O staining. **d**, **e** TC, TG levels of LO2 cells after 20% HTG-medicated serum treatment for 24 h. **f** Red oil O staining of HepG2 cells after 10% HTG-medicated serum treatment for 24 h. Scale bars, 25 μm. **g** TG levels of HepG2 cells after HTG treatment for 24 h. **h**, **i** Pyruvate, lactate levels in LO2 cells after HTG treatment. **j**, **k**, **l** Extracellular acidification rate (ECAR), basal glycosis, and compensatory glycolysis in LO2 cells. *p < 0.05, **p < 0.01, versus FFA group

To evaluate cytotoxicity, LO2 cells (Additional file 4: Fig. S1) were treated with different concentrations of FFA (0, 0.25, 0.5, 1.0, and 1.5 mM) or HTG-medicated serum (0%, 5%, 10%, 15%, and 20%) for 24 h and no disruption in cell viability was detected after intervention with FFA- or HTG-medicated serum (Fig. 3a, b). Compared with the FFA-treated group, lipid droplets were significantly reduced after HTG-medicated serum intervention (Fig. 3c). As expected, the TC and TG levels in LO2 cell lysates were reduced (Fig. 3d, e). Similar results were observed in HepG2 cells, where TG levels reduced and lipid deposition improved (Fig. 3f, g). In addition, elevated lactate and pyruvate levels were detected in LO2 cell supernatant after HTG treatment (Fig. 3h, i). LO2 cells treated with HTG displayed enhanced compensatory glycolysis and showed no differences in ECAR or basal glycolysis (Fig. 3j–l).

### HTG administration enhanced the lactylation in hepatocytes stimulated with FFA

**Fig. 4:**
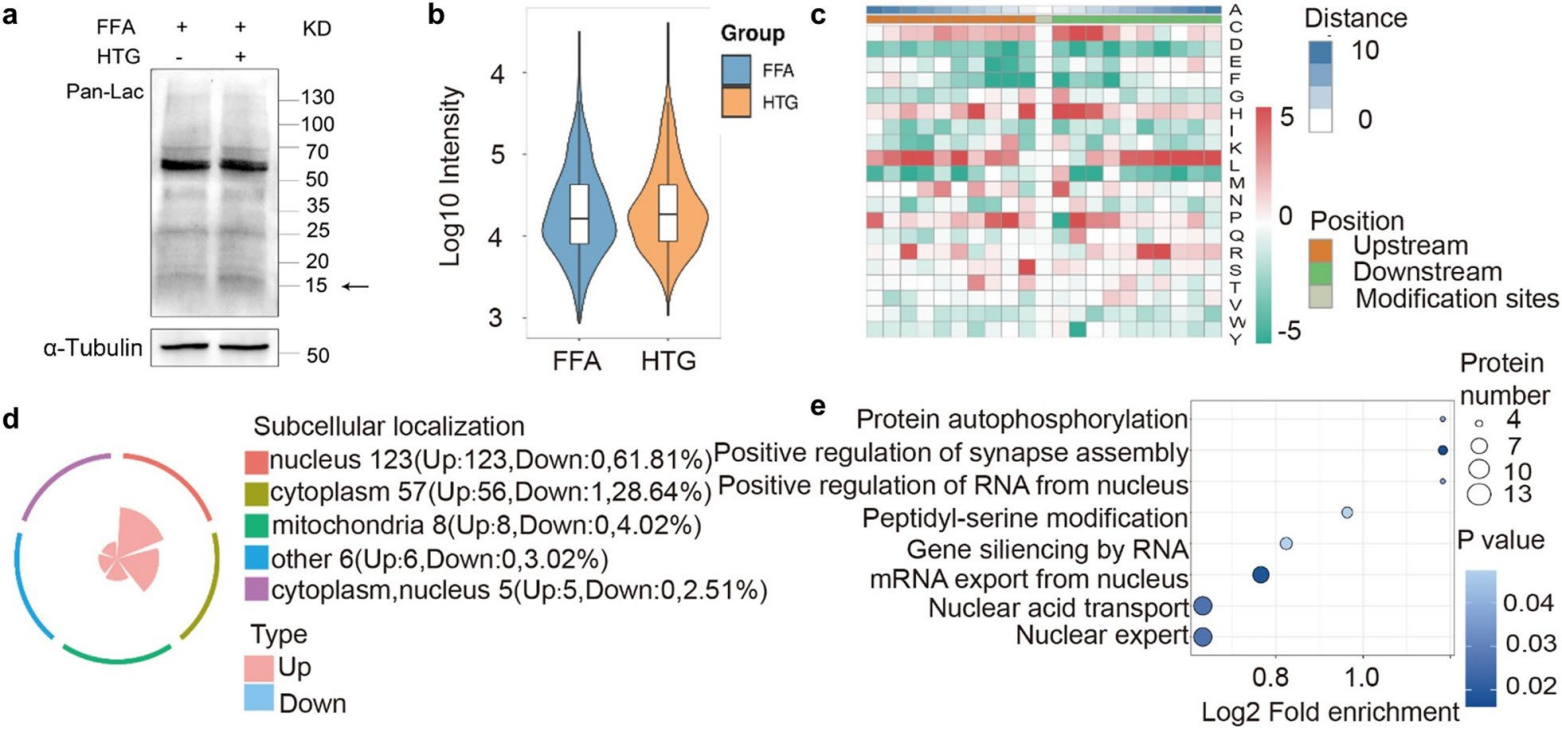
Effects of HTG on protein lactylation. **a** Panlactylated protein expression. **b** Differentially lactylated proteins and modified sites. **c** Heat map of the modification sites near the amino acid. **d** Subcellular localization of lactylated proteins. **e** KEGG pathway analysis

Panlactylated protein was detected (Fig. 4a), and lactylation proteomics was performed in LO2 cells stimulated with FFA after HTG treatment. A total of 199 proteins and 270 lysine lactylation (Kla) sites were identified (Fig. 4b, c), including 1 Kla site on 1 protein was downregulated (fold change < 0.5), 269 Kla sites on 198 proteins were upregulated (fold change > 1.5). The 198 proteins with high lactylation levels after HTG treatment, included histone H2B (K6) and H4 (K80) lactylation. Lactylation links lipid metabolism with gene regulation. The heatmap showed the modification sites near the amino acid frequency change on a scale (DS), which indicates that the frequency of lysine modification sites was significantly increased (Fig. 4c). Subcellular localisation showed that 123 proteins were localised in the nucleus, and 57 proteins were localised in the cytoplasm (Fig. 4d). KEGG pathway enrichment showed that the pathway of RNA-mediated gene silencing was a significant biological process, which indicated that miRNA function played an important role in lactylation after HTG treatment (Fig. 4e).

### HTG administration disrupted miR-155-5p expression in hepatocytes

**Fig. 5:**
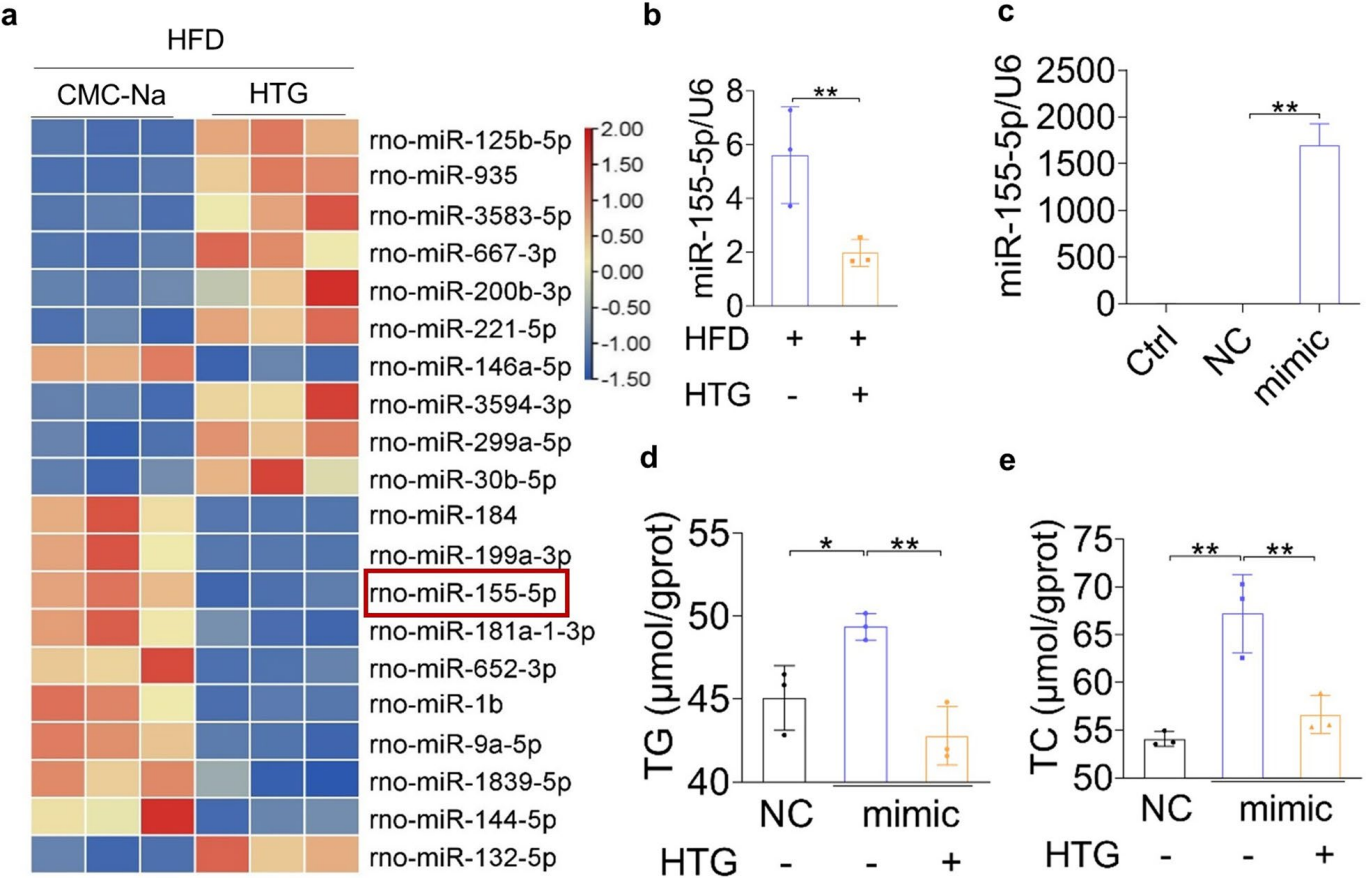
Effects of HTG on liver miRNAs expression. **a** Hierarchical cluster analysis of differentially expressed miRNAs between HFD group and HTG group in dyslipidaemia rats. **b** Relative expression of miR-155-5p detected by RT-PCR. **c** Relative expression of miR-155-5p in LO2 cells after miR-155-5p mimics intervention for 48 h by RT-PCR. **d**, **e** Effects of miR-155-5p mimics on TC, TG levels of LO2 cells stimulated with FFA after 20% HTG-medicated serum treatment for 24 h. *p < 0.05, **p < 0.01, versus HFD group or FFA group

Deep sequencing of miRNAs revealed 119 differentially expressed miRNAs in the HTG group, in which 72 miRNAs were upregulated and 47 miRNAs were downregulated. The top 10 upregulated and downregulated miRNAs compared to the HFD group are shown in Fig. 5a. The expression of rno-miR-155-5p was significantly lower in the HTG group than that in the HFD group (Fig. 5b). To further confirm HTG improved lipid accumulation by downregulating miR-155-5p. miR-155-5p mimics were transfected into LO2 cells, which significantly increased TC and TG levels in LO2 cells (Fig. 5c–e).

### HTG administration enhanced histone lactylation and RNA processing in hepatocytes

**Fig. 6:**
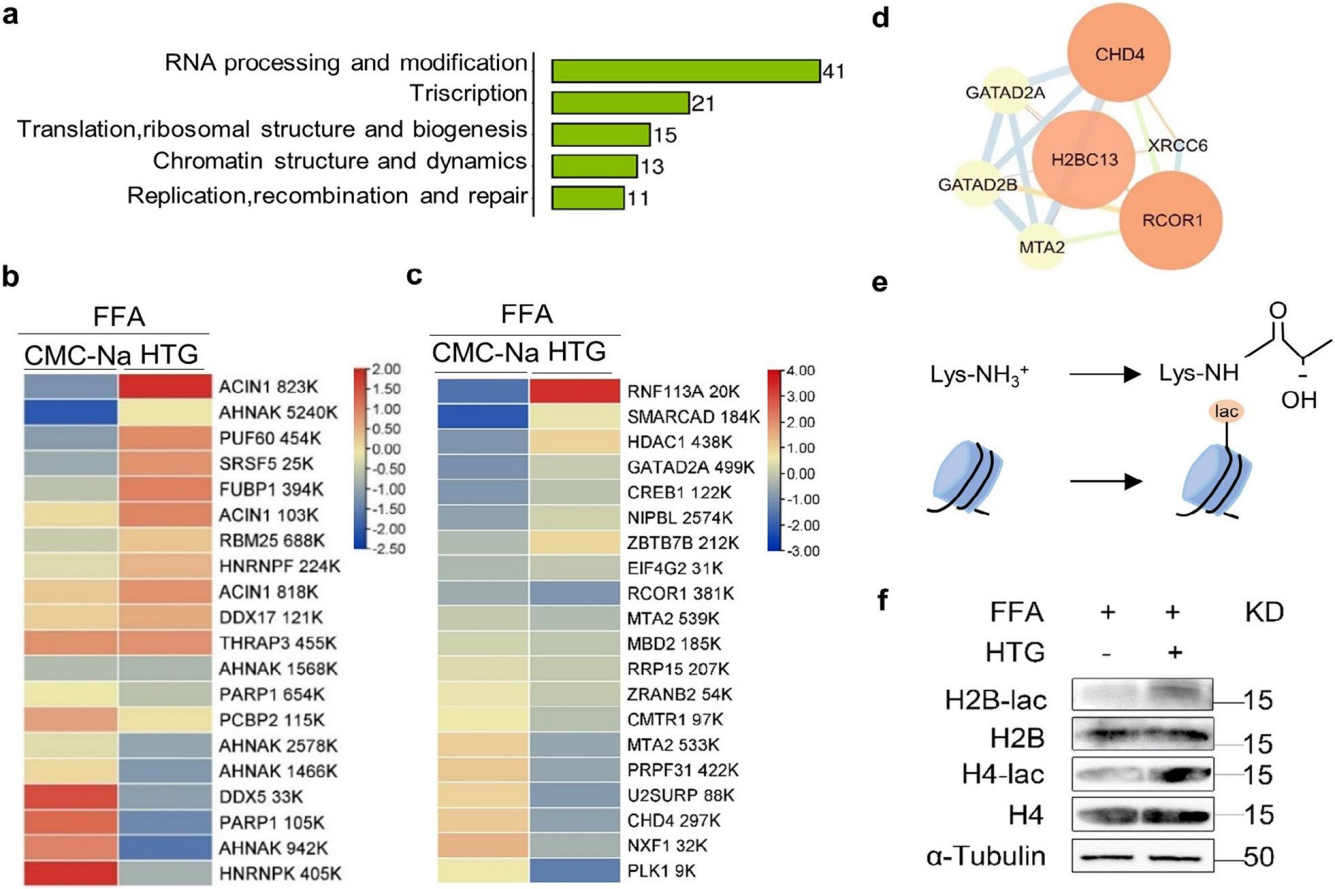
Effects of HTG on histone lactylation. **a** Number of lactylated proteins enriched in genetic information processing via KEGG pathway analysis. **b** Cluster of lactylated proteins involved in regulation of RNA splicing. **c** Cluster of lactylated proteins (top 20) involved in regulation of cellular metabolic process. **d** PPI analysis of H2BC13. **e** Histone lactylation. **f** Western blot analysis of H2B, H4 lactylation levels

The COG/KOG functional classification revealed that 41 proteins were enriched in RNA processing and modification (Fig. 6a). The differentially lactylated proteins which regulate RNA processing, cellular metabolic process were shown in Fig. 6b, c. Histone H2B lactylation strongly correlated with cellular metabolic process, including chromodomain helicase DNA binding protein 4 (CHD4) and REST corepressor 1 (RCOR1) (Fig. 6d, e). Moreover, the expression of histone H2B, and H4 lactylation after HTG treatment in cells stimulated with FFA was significantly upregulated, as detected by western blotting (Fig. 6f).

## Discussion

Dyslipidaemia is a prevalent metabolic disease associated with a high risk of cardiovascular diseases, such as coronary disease and stroke. The effective prevention and management of dyslipidaemia significantly reduces cardiovascular morbidity and mortality [1–3]. Recently, TCM has garnered increasing attention due to its effectiveness and low toxicity in treating dyslipidaemia [12, 13]. In this study, we investigated the efficacy and safety of HTG in regulating lipid metabolism in animal models and in clinical practice. HTG significantly decreased serum levels of TC and LDL-C in dyslipidaemic rats after 8 weeks of treatment. Additionally, HTG reduced the body weight and liver index of rats and improved liver histological changes, especially lipid accumulation. Clinical practice has shown that HTG could significantly lower the concentrations of TC and LDL-C in the plasma of patients with dyslipidaemia, while AST, ALT, BUN, and Cr levels in the serum remained within the normal range after herbal medicine formula treatment.

In this study, we induced dyslipidaemia in rats using a high-fat diet, similar to the dietary habits of patients with hyperlipidaemia. Notably, high-fat diet-induced dyslipidaemia syndrome correlates well with the TCM syndrome of spleen deficiency, phlegm turbidity, and blood turbidity stagnation, whereas the dyslipidaemia model established through glucocorticoid injection represents a kidney-yang deficiency model [14]. HTG is a prescription containing seven herbs, some of which regulate lipid metabolism. For example, hyperoside, a predominant flavonoid in *nelumbinis folium*, has been shown to effectively lower cholesterol and triglyceride levels in rats with non-alcoholic fatty liver disease (NAFLD) via cholesterol metabolism pathways, such as amino acid N-acyltransferase 2 (Acnat2) and Apolipoprotein E (ApoE) [15].

MiRNAs have been widely used as biomarkers for the diagnosis and treatment of various diseases, including dyslipidaemia and atherosclerosis [16]. The liver is a dynamic organ which expresses diverse miRNAs that regulate lipid metabolism [17]. In the present study, we found that HTG restored the liver miRNA profile in dyslipidaemic rats, especially that of rno-miR-155-5p. MiR-155-5p was found to be highly expressed in the liver tissues of NAFLD rats and to promote NAFLD progression [18]. Moreover, miR-155-5p regulates cholesterol efflux and promotes atherosclerosis [19, 20]. Here, we demonstrate that HTG administration disrupts miR-155-5p expression and lipid accumulation in hepatocytes.

Histone post-translational modifications are essential epigenetic processes which regulate chromatin functions and gene transcription, and are closely related to various diseases, including dyslipidaemia and NAFLD [21, 22]. Previous studies have shown that histone modifications (trimethylation, acetylation, etc.) can regulate cellular biological processes by targeting lipid metabolism genes or interfering with miRNA biogenesis [23, 24]. Recently, studies found that lactylation is a novel metabolic reprogramming code, and histone lactylation links to gene transcription and metabolic regulation [11]. It may be crucial to elucidate the mechanism of HTG in treating dyslipidaemia from the perspective of histone lactylation. Here, we found that HTG could increase the concentration of lactate in liver cells, which induced protein lactylation, further analysis revealed that the lactylated proteins enriched in RNA processing or cellular metabolic process. Moreover, we found that the expression of histone H2B, and H4 lactylation upregulated after HTG treatment in liver cells stimulated with FFA. These results indicate that HTG may regulate lipid metabolism by enhancing histone lactylation and downregulating miR-155-5p expression. The relation between histone lactylation and miR-155-5p in regulating lipid metabolism still needs to be further explored. Our study provides a new therapeutic strategy for the treatment of dyslipidaemia.

## Conclusions

HTG is an effective formula for treating dyslipidaemia, especially in lowering TC and LDL-C levels. It is safe, and does not damage liver or kidney function. This potential mechanism may be related to the promotion of lactylation in hepatocytes, and the retardation of miR-155-5p biogenesis.

### Supplementary Information

**Additional file 1: Table S1.** Primer sequences used for qRT-PCR.

**Additional file 2: Table S2.** Baseline characteristics of patients enrolled (n = 12).

**Additional file 2: Table S3.** Liver function, kidney function before and after HTG treatment.

**Additional file 2: Figure S1.** The STR profiling report and authentication certificate of LO2 cells.

## Data Availability

Raw data reported in this article, including RNA-seq, miRNA-seq, have been deposited in the Gene Expression Omnibus database under the accession number GSE241805, GSE241806. The mass spectrometry proteomics data have been deposited to the ProteomeXchange Consortium via the PRIDE [25] partner repository with the dataset identifier PXD045069. All data used and/or analysed in this study are available from the corresponding author on reasonable request.

## Abbreviations

HTG: Huazhuo Tiaozhi granule
TC: Total cholesterol
TG: Triglyceride
LDL-C: Low-density lipoprotein cholesterol
HDL-C: High-density lipoprotein cholesterol
AST: Aspartate transaminase
ALT: Alanine transaminase
TCM: Traditional Chinese medicine
FFA: Free fatty acids
SPF: Specific pathogen free
CMC-Na: Carboxymethyl cellulose-Na
NFD: Normal-fat diet
HFD: High-fat diet
XZK: Xuezhikang
H&E: Hematoxylin and eosin
DMEM: Dulbecco’s modified Eagle’s medium
FBS: Foetal bovine serum
GO: Gene Ontology
KEGG: Kyoto Encyclopedia of Genes and Genomes
qRT-PCR: Quantitative real-time polymerase chain reaction
SD: Sodium dodecyl sulfate
miRNA: MicroRNA
TBST: Tris buffered saline with Tween-20
BUN: Urea nitrogen
Cr: Creatinine
ECAR: Extracellular acidification rate
LC–MS/MS: Liquid chromatography coupled to tandem mass spectrometry
ReSpect: RIKEN MSn spectral database for phytochemicals
GNPS: Global Natural Products Social Molecular Networking
BP: Biological processes
Kla: Lysine lactylation
CHD4: Chromodomain helicase DNA binding protein 4
RCOR1: REST corepressor 1
NAFLD: Non-alcoholic fatty liver disease
Acnat2: Amino acid *N*-acyltransferases 2
ApoE: Apolipoprotein E
DEG: Different expressed gene
