# Supplementary material for: Herbal medicine formula Huazhuo Tiaozhi granule ameliorates dyslipidaemia via regulating histone lactylation and miR-155-5p biogenesis
Source: Clin Epigenetics. 2023 Nov 2;15:175. doi: 10.1186/s13148-023-01573-y (PMC10623728; doi:10.1186/s13148-023-01573-y)
Supplement: Supplementary file 1 — Additional file 1: Table S1. Primer sequences used for qRT-PCR. [file 13148_2023_1573_MOESM1_ESM.docx]

**Additional Files**

**Additional file 1: Table S1.** Primer sequences used for qRT-PCR.

|  | **Primer sequences** |
| --- | --- |
| rno-miR-155-5p | Forward: 5’-TTAATGCTAATTGTGATAGGGGT-3’ |
| U6 | Forward: 5’-CTCGCTTCGGCAGCACATATACT-3’ |
|  | Reverse: 5’-ACGCTTCACGAATTTGCGTGTC-3’ |
| Bulge-Loop hsa-miR-155-5p Primer Set | RiboBio, MQPS0003669-1-100 |
| Bulge-Loop U6 qPCR Primer Set | RiboBio, MQPS0000002-1-100 |
| micrON hsa-miR-155-5p mimic | RiboBio, miR1180504053308-1-5 |
| micrON mimic NC #22 | RiboBio, miR1N0000001-1-5 |
