# Supplementary material for: Herbal medicine formula Huazhuo Tiaozhi granule ameliorates dyslipidaemia via regulating histone lactylation and miR-155-5p biogenesis
Source: Clin Epigenetics. 2023 Nov 2;15:175. doi: 10.1186/s13148-023-01573-y (PMC10623728; doi:10.1186/s13148-023-01573-y)
Supplement: Supplementary file 2 — Additional file 2: Table S2. Baseline characteristics of patients enrolled (n = 12). [file 13148_2023_1573_MOESM2_ESM.docx]

**Additional file 2: Table S2.** Baseline characteristics of patients enrolled (n=12).

|  | **0 week** |
| --- | --- |
| Sex (male/female) | 10/2 |
| Age (years) | 60.00±12.83 |
| BMI (kg/m^2^) | 24.09±3.04 |
