# Supplementary material for: Herbal medicine formula Huazhuo Tiaozhi granule ameliorates dyslipidaemia via regulating histone lactylation and miR-155-5p biogenesis
Source: Clin Epigenetics. 2023 Nov 2;15:175. doi: 10.1186/s13148-023-01573-y (PMC10623728; doi:10.1186/s13148-023-01573-y)
Supplement: Supplementary file 3 — Additional file 2: Table S3. Liver function, kidney function before and after HTG treatment. [file 13148_2023_1573_MOESM3_ESM.docx]

**Additional file 3: Table S3.** Liver function, kidney function before and after HTG treatment.

|  | **0 week** | **8 week** |
| --- | --- | --- |
| ALT（U/L） | 20.51±12.34 | 19.34±8.44 |
| AST（U/L） | 21.69±7.29 | 20.68±6.40 |
| BUN（mmol/L） | 6.45±2.38 | 5.67±1.64 |
| Cr（mmol/L） | 68.43±10.52 | 70.01±11.01 |
