# Supplementary figures and images for: Herbal medicine formula Huazhuo Tiaozhi granule ameliorates dyslipidaemia via regulating histone lactylation and miR-155-5p biogenesis
Source: Clin Epigenetics. 2023 Nov 2;15:175. doi: 10.1186/s13148-023-01573-y (PMC10623728; doi:10.1186/s13148-023-01573-y)

**
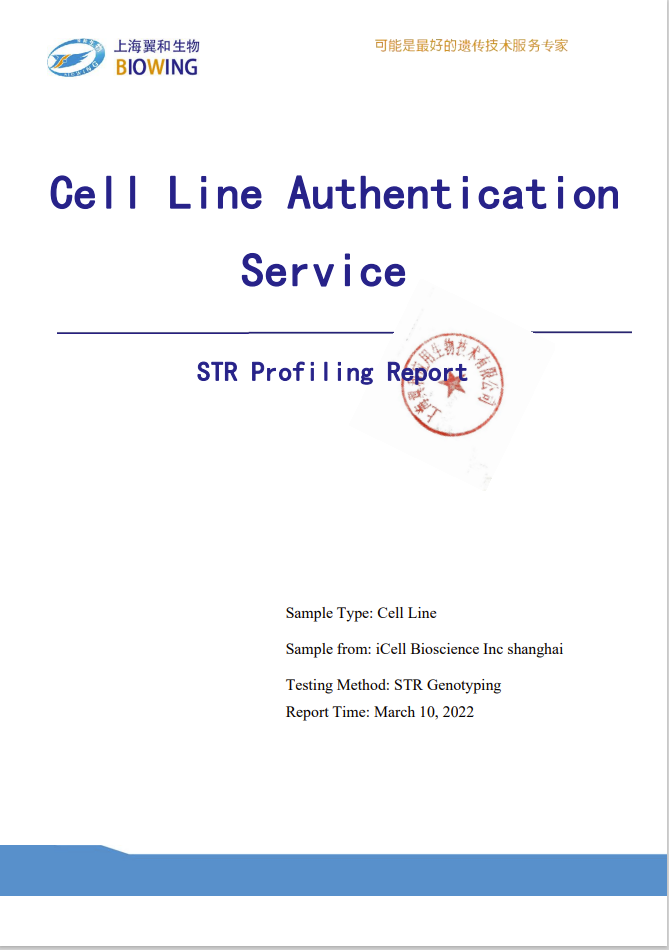
**

**
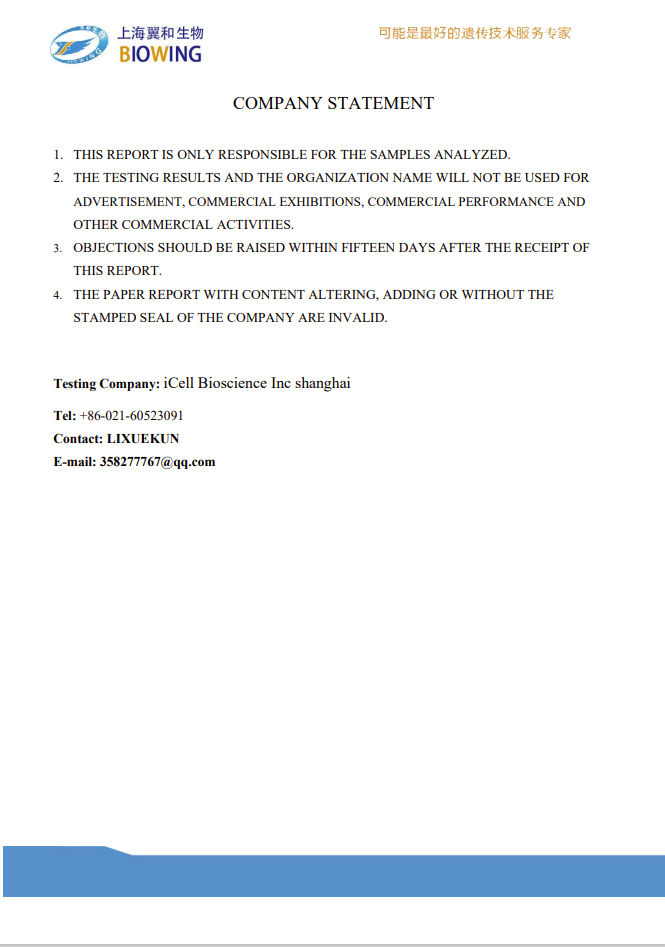
**

**
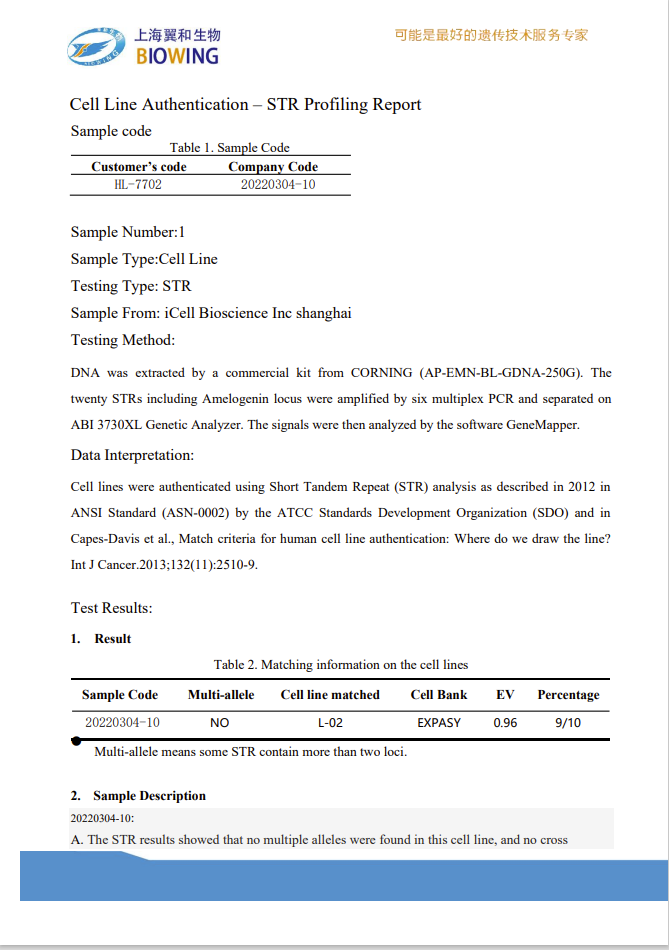
**

**
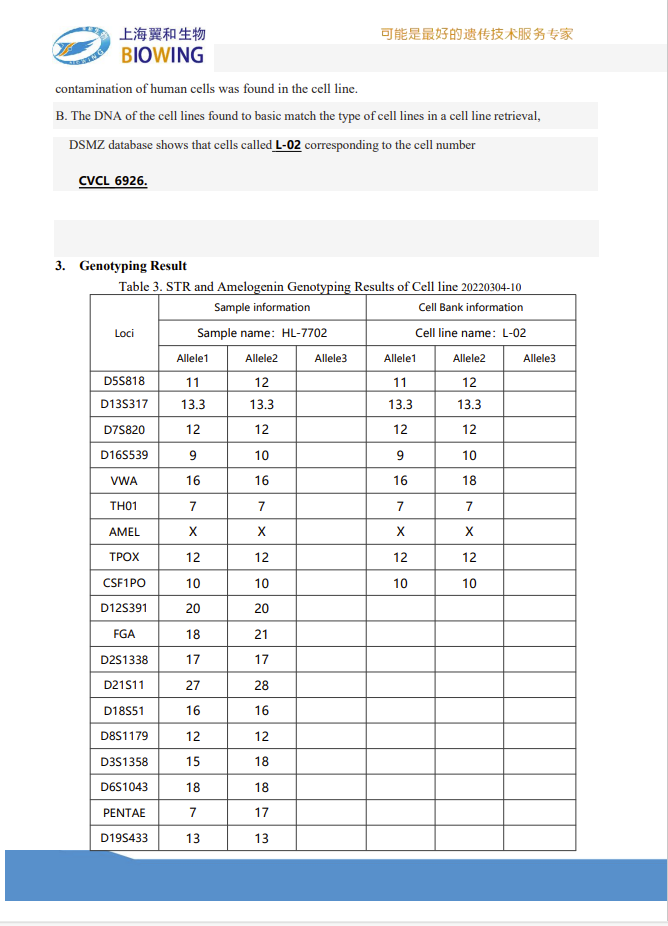
**

**
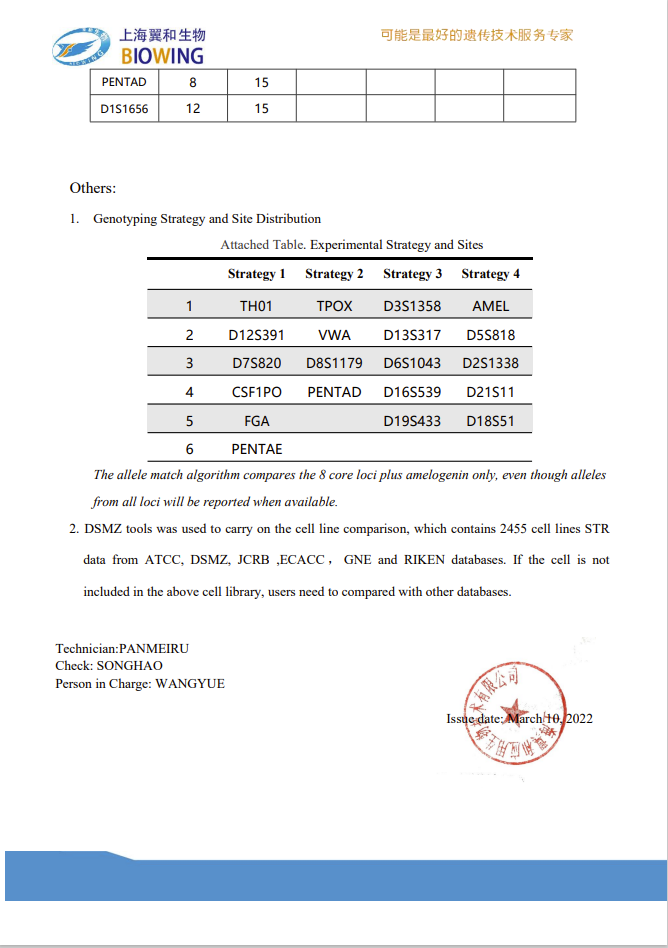
**

**Additional file 4: Figure S1.** The STR profiling report and authentication certificate of LO2 cells.

Supplement: Supplementary file 4 — Additional file 2: Figure S1. The STR profiling report and authentication certificate of LO2 cells. [file 13148_2023_1573_MOESM4_ESM.docx]
